# Perioperative and Long-Term Outcomes of Lower Extremity Revascularization in Patients With Malignancy

**DOI:** 10.1177/15385744261441009

**Published:** 2026-04-08

**Authors:** Lily Cormier, Annie Cherner, Mitchell W. Cox

**Affiliations:** 1 John Sealy School of Medicine, University of Texas Medical Branch, Galveston, TX, USA; 2 Division of Vascular Surgery, Department of Surgery, University of Texas Medical Branch, Galveston, TX, USA

**Keywords:** cancer, chronic limb-threatening ischemia, hypercoagulability, lower extremity bypass, lower extremity arterial angioplasty and stenting

## Abstract

**Objective:**

Cancer patients in general are thought to be poor candidates for lower extremity revascularization procedures due to hypercoagulability, potential need for chemotherapy, or multiple other cancer-related co-morbidities. While vascular surgeons may shy away from these patients, only one previous study we found has assessed the effect of this relationship on lower extremity bypass patency, and substantially worse outcomes were documented. Our study aims to quantify the risk of poor outcomes for lower extremity revascularization in a large sample of cancer patients, utilizing the TriNetX database.

**Methods:**

Within the US cohort of 55 healthcare organizations in the TriNetX database, we identified 1419 patients diagnosed with either breast, prostate, colon, or non-small cell lung cancer of any stage and 60 609 patients without diagnosis of those cancers who had undergone either an open infra-inguinal bypass of any graft type or endovascular intervention on the infra-inguinal arteries from September 3, 2004 to September 3, 2024. Propensity score matching was conducted, which yielded 1413 patients analyzed in each group, and incidence of arterial thrombectomy, bypass revision, amputation, and arteriogram were recorded for each group at 30-day and 1-year time points. Secondary subgroup analyses were conducted within the cancer cohort to determine whether rates of the same outcomes listed above varied between open versus endovascular interventions.

**Results:**

In the primary analysis, there was no significant difference in any of the 4 outcomes between cancer patients and non-cancer patients. In the subgroup analysis, open bypass was found to have a significantly lower incidence of repeat arteriogram within 30 days as compared to endovascular intervention (3.62% vs 7.97%; risk ratio [RR], 0.455, *P* = 0.029) and 1 year (18.84% vs 28.62%; risk ratio [RR], 0.658, *P* = 0.007). All other outcomes in both the 30-day and 1-year follow-up periods for the subgroup analysis did not vary significantly between the 2 groups.

**Conclusion:**

It is possible that patients with a recent diagnosis of cancer may not have worse outcomes for lower extremity bypass compared to patients without a cancer diagnosis. Cancer patients undergoing endovascular interventions, in contrast, did require more repeat interventions which may suggest that open interventions are preferable in this population. We would, however, caution that overall limb salvage was reasonable in this patient population and results were not markedly worse with any intervention strategy. Cancer diagnosis should not necessarily be seen as a contraindication to a lower extremity revascularization and should be considered on a case-by-case basis.

## Article Highlights

### Type of Research

Multicenter retrospective analysis of prospectively collected registry data (TriNetX database).

### Key Findings

Propensity score-matched patients with open or endovascular infra-inguinal revascularization for peripheral arterial disease, with or without a previous diagnosis of cancer in the previous year, demonstrated no significant difference in both perioperative and long-term adverse surgical outcomes. Among patients with cancer, those who underwent open bypass were found to have a lower incidence of subsequent arteriograms compared to those with endovascular interventions.

### Take Home Message

A cancer diagnosis alone should not be a contraindication to either open or endovascular lower extremity arterial revascularization.

## Table of Contents Summary

Multicenter retrospective analysis of prospectively collected registry data which indicated there are no significant differences in perioperative or long-term adverse outcomes of lower extremity revascularization between patients with or without cancer. A cancer diagnosis alone should not be a contraindication to vascular intervention.

## Introduction

With an aging population in the United States, the yearly incidence of many types of malignancy has been on the rise, notably for breast, prostate, and colorectal cancer.^1^ Peripheral arterial disease (PAD) is also common in the elderly population, a problem which may worsen as the prevalence of diabetes increases.^2^ Therefore, it can be inferred that it is increasingly common for vascular surgeons to be making decisions on the treatment of PAD in the context of a recently diagnosed malignancy. Patients with more severe manifestations of PAD may require revascularization either before or during treatment for a malignancy. Non-oncologic surgical interventions in patients with cancer have been a controversial topic due to many factors, such as limited patient life expectancy, adverse cardiac and pulmonary effects from chemotherapy, chemotherapy-induced immunosuppression increasing risk for infection and slowed wound healing.^3^

While a malignancy may increase the complication rate for any procedure, a particularly worrisome risk for the vascular surgeon is hypercoagulability. Trousseau’s syndrome, migratory thrombophlebitis associated with pancreatic cancer, was one of the earliest recognized hypercoagulable syndromes associated with malignancy, but almost any type of cancer can increase risk of both venous (VTE) and arterial thromboembolism (ATE). Whether Trousseau’s syndrome, or hypercoagulability more generally, is due to a hypercoagulable state induced by the cancer cells themselves, downstream effects of certain chemotherapy drugs, or patient immobility from burden of their disease, is not fully known, but is likely a combination of factors. Regardless, complications of VTE or ATE is the second-leading cause of death in cancer patients after the cancer itself, and many surgeons might consider a recent cancer diagnosis to be a relative or absolute contraindication to vascular intervention.^4^ In our literature review, we found one previous study that had investigated the effect of cancer-associated hypercoagulability on lower extremity bypass patency, with significantly worse patency rates documented in patients diagnosed with cancer within a year post-bypass.^5^ In addition, a 2014 study of revascularization in patients with more advanced malignancy demonstrated outcomes that were profoundly poor and recommended against intervention in most or all of these cases.^6^

On the other hand, many patients with cancer are not terminal or have a life expectancy of many years, and severe PAD or limb loss may be debilitating, leading to decreased function, loss of mobility, and a poor quality of life. Ongoing symptoms associated with PAD may even negatively impact cancer treatment, especially if there is ischemic tissue loss. While advanced malignancy is certainly associated with very poor outcomes for vascular surgery procedures, it is an open question whether cancer generally represents a significant risk for revascularization failure. In this study, we would like to document and quantitate the effects of malignancy on the results of lower extremity revascularization. A direct comparison of outcomes between vascular patients with and without a current diagnosis of cancer may change the current narrative around lower extremity revascularization procedures in the setting of a cancer diagnosis and cancer treatment.

## Methods

The data used in this study were collected from the Tri- NetX Global Research Network (TriNetX, LLC, Cambridge, MA). Begun in 2013, this database provides access to deidentified electronic health records for >250 million patients from >120 health care organizations. This database provides continuous, up-to-the-month patient data from 19 countries, predominantly from the United States. The available data includes information on the demographics, diagnoses (based on the International Classification of Diseases, 10th Revision [ICD-10] codes, procedures [coded in the ICD-10-Procedure Coding System] or Current Procedural Terminology [CPT] codes), laboratory tests (coded in Logical Observation Identifiers Names and Codes), medications (coded in Veteran Affairs National Formulary), genomics (coded in Human genome Variation Society), and health care use. Data displayed on the TriNetX Platform, whether in aggregate or at the patient level, contain only deidentified data as per the deidentification standard defined in Section x164.514(a) of the Health Insurance Portability and Accountability Act Privacy Rule. The study used deidentified patient records and did not involve the collection, use, or transmittal of individually identifiable data. The study was given exempt status by the University of Texas Medical Branch at Galveston Institutional Review Board.^7^

We conducted a propensity score-matched retrospective cohort study of adult (≥18 years) patients within the TriNetX US Collaborative Network who underwent infra-inguinal open bypass of any graft type or endovascular revascularization with angioplasty and/or stenting to treat PAD, with or without a current diagnosis of cancer. Using CPT and ICD codes, we identified patients for our experimental cancer cohort who had a diagnosis of either breast, prostate, colorectal, or non-small cell lung cancer of any stage within a year prior to an infra-inguinal open or endovascular arterial revascularization procedure. We chose these particular cancers because they are often treatable and patients may have a significant life expectancy following diagnosis. Of note, cancer stage was not included in this analysis, as it is not available in the majority of TriNet-X records. The time period of data collected was from September 3, 2004 to September 3, 2024. Our final analysis was conducted on September 4, 2024, so it included all data available in the database up to the day before the analysis. We did not choose a specific start date, as we queried the entire TriNetX database, excluding patients with data over 20 years old.

The control non-cancer cohort was identified as patients who underwent the same procedures, but without a diagnosis of those 4 select cancers in the year leading up to their intervention. Both cohorts were also required to have a diagnosis of chronic limb-threatening ischemia (CLTI) prior to intervention, which included claudication, rest pain, and/or tissue loss. Cohorts were matched by age, gender, race, hypertension, diabetes, pulmonary heart disease and diseases of pulmonary circulation, ischemic heart diseases, cerebrovascular diseases, and chronic kidney disease status. Other variables such as prescribed antilipemic agents, anticoagulants, and platelet aggregation inhibitors at the time of intervention were also controlled for.

Outcomes were based on the incidence of specific perioperative and long-term complications after revascularization. Outcomes investigated at both 30 days and 1 year post-operatively included arterial thrombectomy, bypass revision, major and minor amputation, and repeat arteriogram. A secondary subgroup analysis was also conducted within the experimental cancer cohort, which compared the same 4 outcomes, at both 30 days and 1 year, between those who had open vs endovascular interventions. Propensity matching with the same variables mentioned above was also performed for the secondary analysis. All outcomes, both perioperative and long-term, were analyzed as a risk ratio (RR) with 95% confidence interval (CI).

## Results

Based on these criteria, our primary analysis included 60 609 patients in the non-cancer cohort and 1419 patients in the cancer cohort. Propensity score matching was then performed to yield 1413 patients from each group. The CPT and ICD-10 codes that were used in this study as well as the full query for each patient cohort and codes for the outcomes are summarized in Supplemental Tables I, II, and III (online only). The matched comorbidities and demographics are shown in Table 1 and were comparable between the 2 groups.^7^ In both the perioperative and long-term period, there was no significant difference in any of the 4 outcomes between cohorts (Tables 2 and 3).

**Table 1.** Characteristics of Patients Undergoing Lower Extremity Revascularization Procedures for CLTI

Covariate results	Before propensity score matching			After propensity score matching		
	Cancer (n = 1419)	No cancer (n = 60 609)	*P* value	Cancer (n = 1413)	No cancer (n = 1413)	*P* value
Age, years	70.0 ± 8.5	66.8 ± 10.4	<0.001	70.0 ± 8.5	69.9 ± 8.7	0.960
Male sex	894 (63.3)	36 013 (59.8)	0.008	894 (63.3)	901 (63.8)	0.784
Caucasian race	961 (68.0)	39 332 (65.3)	0.035	961 (68.0)	999 (70.7)	0.058
Hypertension	691 (48.9)	25 644 (42.6)	<0.001	691 (48.9)	688 (48.7)	0.910
Cerebrovascular diseases	206 (14.6)	6599 (11.0)	<0.001	206 (14.6)	191 (13.5)	0.417
Pulmonary heart disease & diseases of pulmonary circulation	73 (5.2)	2335 (3.9)	0.013	73 (5.2)	65 (4.6)	0.485
Ischemic heart diseases	513 (36.3)	18 883 (31.4)	<0.001	513 (36.3)	518 (36.7)	0.845
Diabetes	542 (38.4)	25 933 (43.1)	<0.001	542 (38.4)	562 (39.8)	0.441
Chronic kidney disease	304 (21.5)	13 365 (22.2)	0.545	304 (21.5)	285 (20.2)	0.379
Antilipemic agents	585 (41.4)	22 239 (36.9)	0.001	585 (41.4)	588 (41.6)	0.909
Anticoagulants	614 (43.5)	23 116 (38.4)	<0.001	614 (43.5)	630 (44.6)	0.544
Platelet aggregation inhibitors	587 (41.5)	22 017 (36.6)	<0.001	587 (41.5)	599 (42.4)	0.647

Results given as number ± standard deviation or n (%).

**Table 2.** Propensity-Score Matched Perioperative Outcomes From Primary Analysis

	Cancer (n = 1413)	No cancer (n = 1413)	RR [95% CI]
Arterial thrombectomy	≤10 (0.7)	≤10 (0.7)	1 (0.418, 2.395)
Bypass revision	≤10 (0.7)	11 (0.8)	0.909 (0.387, 2.134)
Amputation	133 (9.4)	149 (10.5)	0.893 (0.750, 1.114)
Repeat arteriogram	87 (6.2)	108 (7.6)	0.806 (0.613, 1.058)

*CI*, confidence interval; *RR,* risk ratio.

Results given as number (%).

**Table 3.** Propensity-Score Matched Long-Term Outcomes From Primary Analysis

	Cancer (n = 1413)	No cancer (n = 1413)	RR [95% CI]
Arterial thrombectomy	13 (0.9)	19 (1.3)	0.684 (0.339, 1.380)
Bypass revision	17 (1.2)	20 (1.4)	0.85 (0.447, 1.616)
Amputation	242 (17.1)	264 (18.7)	0.917 (0.783, 1.074)
Repeat arteriogram	349 (24.7)	337 (23.0)	1.036 (0.909, 1.180)

*CI*, confidence interval; *RR,* risk ratio.

Results given as number (%).

For our secondary subgroup analysis within the cancer cohort, we included 283 patients in the open cohort and 1202 patients in the endovascular cohort. Propensity score matching was then performed to yield 276 patients from each group. Open bypass was found to have a significantly lower incidence of repeat arteriogram in both the perioperative (3.62% vs 7.97%; risk ratio [RR], 0.455, *P* = 0.029) and long-term time points (18.84% vs 28.62%; risk ratio [RR], 0.658, *P* = 0.007). All other outcomes in both the perioperative and long-term periods did not vary significantly between the 2 groups (Tables 4 and 5).

**Table 4.** Propensity-Score Matched Perioperative Outcomes From Secondary Analysis Within Cancer Cohort

	Open bypass (n = 276)	Endovascular (n = 276)	RR [95% CI]
Arterial thrombectomy	≤10 (3.6)	≤10 (3.6)	1 (0.423, 2.364)
Bypass revision	≤10 (3.6)	≤10 (3.6)	1 (0.423, 2.364)
Amputation	33 (12.0)	30 (10.9)	1.1 (0.691, 1.752)
Repeat arteriogram	≤10 (3.6)	22 (8.0)	0.455 (0.219, 0.942)

*CI*, confidence interval; *RR,* risk ratio.

Results given as number (%).

**Table 5.** Propensity-Score Matched Long-Term Outcomes From Secondary Analysis Within Cancer Cohort

	Open bypass (n = 276)	Endovascular (n = 276)	RR [95% CI]
Arterial thrombectomy	≤10 (3.6)	≤10 (3.6)	1 (0.423, 2.364)
Bypass revision	≤10 (3.6)	≤10 (3.6)	1 (0.423, 2.364)
Amputation	53 (19.2)	56 (20.3)	0.946 (0.676, 1.325)
Repeat arteriogram	52 (18.8)	79 (28.6)	0.658 (0.484, 0.895)

*CI*, confidence interval; *RR,* risk ratio.

Results given as number (%).

## Discussion

This contemporary retrospective cohort study aimed to compare the perioperative and long-term outcomes of open and endovascular lower extremity revascularization procedures in patients with or without a recent diagnosis of cancer. Although cancer patients are traditionally considered a high-risk population in the context of vascular surgery due to hypercoagulability, a diagnosis of cancer within one year prior to intervention was not found to be a risk factor for postoperative complications generally.^8^ Although interestingly, our subgroup analysis within the cancer cohort demonstrated that patients undergoing open bypass surgery had a significantly lower incidence of repeat postoperative arteriograms compared to those undergoing endovascular procedures. Presumably, these are related to re-occlusion or restenosis post-procedure in the endovascular cohort. This finding is not entirely unexpected given recent finding in the BEST trial, although there is continuing controversy in the literature regarding whether patency rates differ between endovascular and open revascularization for CLTI.^9,10^ While this finding may imply superiority of open procedures in cancer patients, it is still essential to balance these potential benefits against the invasiveness and perioperative risks associated with open surgery, particularly when patients may require chemotherapy, radiation, or surgery related to the malignancy. The overall limb salvage rates were favorable across both intervention types, further reinforcing the feasibility of pursuing revascularization in cancer patients when clinically indicated. Our results emphasize that cancer diagnosis alone should not dissuade clinicians from offering potentially limb-salvage procedures. The decision-making surrounding revascularization can be similar to patients without cancer, with considerations given to chemotherapy and radiation schedules, and shared decision-making with the oncology team.

This research is not without limitations due to its retrospective nature and reliance on administrative coding, which lacks clinical nuance that could further illuminate the true effects of cancer on vascular surgical outcomes. Our study is based on a large, multicenter database and lacks patient-level data, including the exact follow-up period, socioeconomic factors, cancer staging, cancer treatment regimens, and the presentation of each patient. It is probable that most of the cancer cohort represented patients who were more ideal surgical candidates at the time of intervention with lower-stage malignancies and better life expectancy. We chose 4 types of cancer to analyze (breast, prostate, colorectal, non-small cell lung) that are generally more common and have regulated screening protocols, such as mammography, PSA surveillance, colonoscopy, and computed tomography. Therefore, a larger proportion of patients diagnosed with these cancers may have had their cancer detected in a much earlier stage and presented for surgery in a healthier state than the average cancer patient.

The lack of nuance in the CPT codes used for measuring outcomes also effects the validity of our study conclusions, as there is no feasible way on TriNetX to confirm that an outcome was specifically related to the index intervention. For example, it is not possible to know whether a postoperative amputation was a direct result of a failure of the index procedure, or even if it was of the same laterality. Similarly, an arterial thrombectomy outcome could not be confirmed to be a thrombectomy of the specific artery that was intervened on and could possibly have been due to a separate acute thrombo-occlusive event. More nuanced studies comparing the effects of different chemotherapy regimens or cancer stages on postoperative intervention patency are warranted to better delineate which subsets of cancer patients make the most ideal surgical candidates. We would, however, advise that cancer diagnosis alone does not necessarily portend a poor outcome for lower extremity revascularization and should not be considered a contraindication for bypass or endovascular intervention.

## Conclusion

Patients with a recent diagnosis of cancer may not have worse outcomes for lower extremity revascularization compared to patients without a cancer diagnosis, indicating cancer itself may not be a contraindication to vascular surgical interventions and should be considered on a case-by-case basis.

## Supplemental Material

Supplemental Material - Perioperative and Long-Term Outcomes of Lower Extremity Revascularization in Patients With Malignancy Supplemental Material for Perioperative and Long-Term Outcomes of Lower Extremity Revascularization in Patients With Malignancy by Lily Cormier, Annie Cherner, Mitchell W. Cox in Vascular and Endovascular Surgery
