# Supplementary material for: Perioperative and Long-Term Outcomes of Lower Extremity Revascularization in Patients With Malignancy
Source: Vasc Endovascular Surg. 2026 Apr 8;60(6):473–8. doi: 10.1177/15385744261441009 (PMC13310305; doi:10.1177/15385744261441009)
Supplement: Supplemental Material - Perioperative and Long-Term Outcomes of Lower Extremity Revascularization in Patients With Malignancy [file sj-pdf-1-ves-10.1177_15385744261441009.pdf]

Supplemental Table I: Query criteria for experimental cohort
<Patients with cancer>

**Ungrouped terms**					
	must have		demographics	Age	Age (at least 18 years (most recent occurrence))
**Group 1**					
	**Group 1A Cancer Dx**				
	must have	any of	diagnosis	UMLS:ICD10CM:C50	Malignant neoplasm of breast (between 18 and 90 years old at event)
			diagnosis	UMLS:ICD10CM:C61	Malignant neoplasm of prostate (between 18 and 90 years old at event)
			diagnosis	UMLS:ICD10CM:C18	Malignant neoplasm of colon (between 18 and 90 years old at event)
			diagnosis	UMLS:ICD10CM:C34	Malignant neoplasm of bronchus and lung
	date constraint		The terms in this group occurred at any time		
	event relationship		Any instance of Bypass/Revascularization occurred within 1 year on or after the first instance of Cancer Dx		
	**Group 1B Bypass/Revascularization**				
	must have	any of	procedure	UMLS:CPT:1020568	Revascularization, endovascular, open or percutaneous, tibial, peroneal artery, unilateral, initial vessel
			procedure	UMLS:CPT:1020569	Revascularization, endovascular, open or percutaneous, tibial/peroneal artery, unilateral, each additional vessel
			procedure	UMLS:CPT:35556	Bypass graft, with vein; femoral-popliteal
			procedure	UMLS:CPT:35558	Bypass graft, with vein; femoral-femoral
			procedure	UMLS:CPT:35566	Bypass graft, with vein; femoral-anterior tibial, posterior tibial, peroneal artery or other distal vessels
			procedure	UMLS:CPT:35570	Bypass graft, with vein; tibial-tibial, peroneal-tibial, or tibial/peroneal trunk-tibial
			procedure	UMLS:CPT:35571	Bypass graft, with vein; popliteal-tibial, -peroneal artery or other distal vessels
			procedure	UMLS:CPT:35583	In-situ vein bypass; femoral-popliteal
			procedure	UMLS:CPT:35585	In-situ vein bypass; femoral-

					anterior tibial, posterior tibial, or peroneal artery
			procedure	UMLS:CPT:35587	In-situ vein bypass; popliteal-tibial, peroneal
			procedure	UMLS:CPT:35661	Bypass graft, with other than vein; femoral-femoral
			procedure	UMLS:CPT:35666	Bypass graft, with other than vein; femoral-anterior tibial, posterior tibial, or peroneal artery
			procedure	UMLS:CPT:35671	Bypass graft, with other than vein; popliteal-tibial or -peroneal artery
			procedure	UMLS:CPT:1020567	Revascularization, endovascular, open or percutaneous, femoral, popliteal artery(s), unilateral
**Group 2**					
	**Group 2A CLTI + Claudication**				
	must have	any of	diagnosis	UMLS:ICD10CM:I70.211	Atherosclerosis of native arteries of extremities with intermittent claudication, right leg (at least 18 years old at event)
			diagnosis	UMLS:ICD10CM:I70.212	Atherosclerosis of native arteries of extremities with intermittent claudication, left leg (at least 18 years old at event)
			diagnosis	UMLS:ICD10CM:I70.213	Atherosclerosis of native arteries of extremities with intermittent claudication, bilateral legs (at least 18 years old at event)
			diagnosis	UMLS:ICD10CM:I70.22	Atherosclerosis of native arteries of extremities with rest pain (at least 18 years old at event)
			diagnosis	UMLS:ICD10CM:I70.23	Atherosclerosis of native arteries of right leg with ulceration (at least 18 years old at event)
			diagnosis	UMLS:ICD10CM:I70.24	Atherosclerosis of native arteries of left leg with ulceration (at least 18 years old at event)
			diagnosis	UMLS:ICD10CM:I70.26	Atherosclerosis of native arteries of extremities with gangrene (at least 18 years old

					at event)
	date constraint		The terms in this group occurred at any time		
	event relationship		Any instance of Bypass/Revascularization occurred on or after any instance of CLTI + Claudication		
	**Group 2B Bypass/Revascularization**				
	must have	any of	procedure	UMLS:CPT:1020568	Revascularization, endovascular, open or percutaneous, tibial, peroneal artery, unilateral, initial vessel
			procedure	UMLS:CPT:1020569	Revascularization, endovascular, open or percutaneous, tibial/peroneal artery, unilateral, each additional vessel
			procedure	UMLS:CPT:35556	Bypass graft, with vein; femoral-popliteal
			procedure	UMLS:CPT:35558	Bypass graft, with vein; femoral-femoral
			procedure	UMLS:CPT:35566	Bypass graft, with vein; femoral-anterior tibial, posterior tibial, peroneal artery or other distal vessels
			procedure	UMLS:CPT:35570	Bypass graft, with vein; tibial-tibial, peroneal-tibial, or tibial/peroneal trunk-tibial
			procedure	UMLS:CPT:35571	Bypass graft, with vein; popliteal-tibial, -peroneal artery or other distal vessels
			procedure	UMLS:CPT:35583	In-situ vein bypass; femoral-popliteal
			procedure	UMLS:CPT:35585	In-situ vein bypass; femoral-anterior tibial, posterior tibial, or peroneal artery
			procedure	UMLS:CPT:35587	In-situ vein bypass; popliteal-tibial, peroneal
			procedure	UMLS:CPT:35661	Bypass graft, with other than vein; femoral-femoral
			procedure	UMLS:CPT:35666	Bypass graft, with other than vein; femoral-anterior tibial, posterior tibial, or peroneal artery
			procedure	UMLS:CPT:35671	Bypass graft, with other than vein; popliteal-tibial or -peroneal artery
			procedure	UMLS:CPT:1020567	Revascularization, endovascular, open or percutaneous, femoral, popliteal artery(s), unilateral

Supplemental Table II: Query criteria for control cohort

<Patients without cancer>

**Ungrouped terms**					
	must have		demographics	Age	Age (at least 18 years (most recent occurrence))
**Group 1**					
	**Group 1A PAD Interventions without Cancer**				
	must have	any of	procedure	UMLS:CPT:1020567	Revascularization, endovascular, open or percutaneous, femoral, popliteal artery(s), unilateral
			procedure	UMLS:CPT:1020568	Revascularization, endovascular, open or percutaneous, tibial, peroneal artery, unilateral, initial vessel
			procedure	UMLS:CPT:1020569	Revascularization, endovascular, open or percutaneous, tibial/peroneal artery, unilateral, each additional vessel
			procedure	UMLS:CPT:35556	Bypass graft, with vein; femoral-popliteal
			procedure	UMLS:CPT:35558	Bypass graft, with vein; femoral-femoral
			procedure	UMLS:CPT:35566	Bypass graft, with vein; femoral-anterior tibial, posterior tibial, peroneal artery or other distal vessels
			procedure	UMLS:CPT:35570	Bypass graft, with vein; tibial-tibial, peroneal-tibial, or tibial/peroneal trunk-tibial
			procedure	UMLS:CPT:35571	Bypass graft, with vein; popliteal-tibial, -peroneal artery or other distal vessels
			procedure	UMLS:CPT:35583	In-situ vein bypass; femoral-popliteal
			procedure	UMLS:CPT:35585	In-situ vein bypass; femoral-anterior tibial, posterior tibial, or peroneal artery
			procedure	UMLS:CPT:35587	In-situ vein bypass; popliteal-tibial, peroneal
			procedure	UMLS:CPT:35661	Bypass graft, with other than vein; femoral-femoral
			procedure	UMLS:CPT:35666	Bypass graft, with other than vein; femoral-anterior tibial, posterior tibial, or peroneal artery
			procedure	UMLS:CPT:35671	Bypass graft, with other than vein; popliteal-tibial or -peroneal artery

	date constraint		The terms in this group occurred at any time		
	event relationship		Any instance of Cancer Dx occurred on or after any instance of PAD Interventions without Cancer		
	**Group 1B Cancer Dx**				
	cannot have		diagnosis	UMLS:ICD10CM:C50	Malignant neoplasm of breast (between 18 and 90 years old at event)
		or	diagnosis	UMLS:ICD10CM:C61	Malignant neoplasm of prostate (between 18 and 90 years old at event)
		or	diagnosis	UMLS:ICD10CM:C34	Malignant neoplasm of bronchus and lung
		or	diagnosis	UMLS:ICD10CM:C18	Malignant neoplasm of colon (between 18 and 90 years old at event)
**Group 2**					
	**Group 2A CLTI + Claudication**				
	must have	any of	diagnosis	UMLS:ICD10CM:I70.211	Atherosclerosis of native arteries of extremities with intermittent claudication, right leg (at least 18 years old at event)
			diagnosis	UMLS:ICD10CM:I70.212	Atherosclerosis of native arteries of extremities with intermittent claudication, left leg (at least 18 years old at event)
			diagnosis	UMLS:ICD10CM:I70.213	Atherosclerosis of native arteries of extremities with intermittent claudication, bilateral legs (at least 18 years old at event)
			diagnosis	UMLS:ICD10CM:I70.22	Atherosclerosis of native arteries of extremities with rest pain (at least 18 years old at event)
			diagnosis	UMLS:ICD10CM:I70.23	Atherosclerosis of native arteries of right leg with ulceration (at least 18 years old at event)
			diagnosis	UMLS:ICD10CM:I70.24	Atherosclerosis of native arteries of left leg with ulceration (at least 18 years old at event)
			diagnosis	UMLS:ICD10CM:I70.26	Atherosclerosis of native arteries of extremities with gangrene (at least 18 years old at event)
	date constraint		The terms in this group occurred at any time		

	event relationship		Any instance of PAD Interventions without Cancer occurred on or after any instance of CLTI + Claudication		
	**Group 2B PAD Interventions without Cancer**				
	must have	any of	procedure	UMLS:CPT:1020567	Revascularization, endovascular, open or percutaneous, femoral, popliteal artery(s), unilateral
			procedure	UMLS:CPT:1020568	Revascularization, endovascular, open or percutaneous, tibial, peroneal artery, unilateral, initial vessel
			procedure	UMLS:CPT:1020569	Revascularization, endovascular, open or percutaneous, tibial/peroneal artery, unilateral, each additional vessel
			procedure	UMLS:CPT:35556	Bypass graft, with vein; femoral-popliteal
			procedure	UMLS:CPT:35558	Bypass graft, with vein; femoral-femoral
			procedure	UMLS:CPT:35566	Bypass graft, with vein; femoral-anterior tibial, posterior tibial, peroneal artery or other distal vessels
			procedure	UMLS:CPT:35570	Bypass graft, with vein; tibial-tibial, peroneal-tibial, or tibial/peroneal trunk-tibial
			procedure	UMLS:CPT:35571	Bypass graft, with vein; popliteal-tibial, -peroneal artery or other distal vessels
			procedure	UMLS:CPT:35583	In-situ vein bypass; femoral-popliteal
			procedure	UMLS:CPT:35585	In-situ vein bypass; femoral-anterior tibial, posterior tibial, or peroneal artery
			procedure	UMLS:CPT:35587	In-situ vein bypass; popliteal-tibial, peroneal
			procedure	UMLS:CPT:35661	Bypass graft, with other than vein; femoral-femoral
			procedure	UMLS:CPT:35666	Bypass graft, with other than vein; femoral-anterior tibial, posterior tibial, or peroneal artery
			procedure	UMLS:CPT:35671	Bypass graft, with other than vein; popliteal-tibial or -peroneal artery

Supplemental Table III: Codes for perioperative and long-term outcomes

**Arterial Thrombectomy**	
	**Outcome definition**

**Arterial Thrombectomy**			
	**Outcome definition**		
	Procedure	UMLS:CPT:1014621	Thrombectomy of arterial or venous graft (other than hemodialysis graft or fistula)
	Procedure	UMLS:CPT:35876	Thrombectomy of arterial or venous graft (other than hemodialysis graft or fistula); with revision of arterial or venous graft
	**Settings for the performed analyses**		
	Risk analysis		including patients with outcome prior to the time window
	Kaplan - Meier survival analysis		including patients with outcome prior to the time window
**Bypass Revision**			
	**Outcome definition**		
	Procedure	UMLS:CPT:1006611	Revision, lower extremity arterial bypass, without thrombectomy, open
	Procedure	UMLS:CPT:35151	Direct repair of aneurysm, pseudoaneurysm, or excision (partial or total) and graft insertion, with or without patch graft; for aneurysm, pseudoaneurysm, and associated occlusive disease, popliteal artery
	Procedure	UMLS:CPT:35131	Direct repair of aneurysm, pseudoaneurysm, or excision (partial or total) and graft insertion, with or without patch graft; for aneurysm, pseudoaneurysm, and associated occlusive disease, iliac artery (common, hypogastric, external)
	Procedure	UMLS:CPT:35131	Direct repair of aneurysm, pseudoaneurysm, or excision (partial or total) and graft insertion, with or without patch graft; for aneurysm, pseudoaneurysm, and associated occlusive disease, iliac artery (common, hypogastric, external)
	Procedure	UMLS:CPT:35879	Revision, lower extremity arterial bypass, without thrombectomy, open; with vein patch angioplasty
	Procedure	UMLS:CPT:35881	Revision, lower extremity arterial bypass, without thrombectomy, open; with segmental vein interposition
	Procedure	UMLS:CPT:35703	Exploration not followed by surgical repair, artery; lower extremity (eg, common femoral, deep femoral, superficial femoral, popliteal, tibial, peroneal)
	Procedure	UMLS:CPT:35860	Exploration for postoperative hemorrhage, thrombosis or infection; extremity
	Procedure	UMLS:CPT:35703	Exploration not followed by surgical repair, artery; lower extremity (eg, common femoral, deep femoral, superficial femoral, popliteal, tibial, peroneal)
	**Settings for the performed analyses**		
	Risk analysis		including patients with outcome prior to the time window
	Kaplan - Meier survival analysis		including patients with outcome prior to the time window
**Amputation**			

**Arterial Thrombectomy**			
	**Outcome definition**		
	**Outcome definition**		
	Procedure	UMLS:CPT:1005524	Amputation Procedures on the Foot and Toes
	Procedure	UMLS:CPT:1005298	Amputation Procedures on the Leg (Tibia and Fibula) and Ankle Joint
	Procedure	UMLS:CPT:1005146	Amputation Procedures on the Femur (Thigh Region) and Knee Joint
	**Settings for the performed analyses**		
	Risk analysis		including patients with outcome prior to the time window
	Kaplan - Meier survival analysis		including patients with outcome prior to the time window
**Arteriogram**			
	**Outcome definition**		
	Procedure	UMLS:CPT:1020567	Revascularization, endovascular, open or percutaneous, femoral, popliteal artery(s), unilateral
	Procedure	UMLS:CPT:1020568	Revascularization, endovascular, open or percutaneous, tibial, peroneal artery, unilateral, initial vessel
	Procedure	UMLS:CPT:1020569	Revascularization, endovascular, open or percutaneous, tibial/peroneal artery, unilateral, each additional vessel
	**Settings for the performed analyses**		
	Risk analysis		including patients with outcome prior to the time window
	Kaplan - Meier survival analysis		including patients with outcome prior to the time window
